# Recurrence patterns in pediatric intracranial ependymal neoplasm: a systematic imaging work-up

**DOI:** 10.1007/s00234-025-03553-w

**Published:** 2025-02-17

**Authors:** Annika Stock, Judith Krumma, Gudrun Fleischhack, Stephan Tippelt, Lydia Rink, Torsten Pietsch, Martin Mynarek, Denise Obrecht-Sturm, Stefan Rutkowski, Stefan M. Pfister, Dominik Sturm, Kristian W. Pajtler, Ulrich Schüller, Beate Timmermann, Rolf-Dieter Kortmann, Brigitte Bison, Mirko Pham, Monika Warmuth-Metz

**Affiliations:** 1 Department of Neuroradiology, University Hospital Wuerzburg, Wuerzburg, Germany; 2 Neuroradiological Reference Center for the Pediatric Brain Tumor (HIT) Studies of the German Society of Pediatric Oncology and Hematology, University Hospital Wuerzburg (until 2020), Wuerzburg, Germany; 3 Diagnostic and Interventional Neuroradiology, Faculty of Medicine, University of Augsburg, (since 2021), Augsburg, Germany; 4 Pediatrics III, Center of Translational Neuro- and Behavioral Sciences (CTNBS), University Medicine of Essen, Essen, Germany; 5 Institute of Neuropathology, DGNN Brain Tumor Reference Center, University of Bonn Medical Center, Bonn, Germany; 6 Department of Pediatric Hematology and Oncology, University Medical Center Hamburg-Eppendorf, Hamburg, Germany; 7 Mildred Scheel Cancer Career Center HaTriCS4, University Medical Center Hamburg-Eppendorf, Hamburg, Germany; 8 Hopp Children’s Cancer Heidelberg (KiTZ), Heidelberg, Germany; 9 Division of Pediatric Glioma Research, German Cancer Consortium (DKTK), German Cancer Research Center (DKFZ), Heidelberg, Germany; 10 Department of Pediatric Hematology and Oncology, Heidelberg University Hospital, Heidelberg, Germany; 11 Institute of Neuropathology, University Medical Center Hamburg-Eppendorf, Hamburg, Germany; 12 Research Institute Children’s Cancer Center Hamburg, Hamburg, Germany; 13 Department of Particle Therapy, West German Proton Therapy Centre Essen (WPE), University Hospital Essen, West German Cancer Centre (WTZ), German Cancer Consortium (DKTK), Essen, Germany; 14 Department of Radiation Oncology, University Leipzig, Leipzig, Germany; 15 Diagnostic and Interventional Neuroradiology, Faculty of Medicine, University of Augsburg, Augsburg, Germany; 16 Department of Pediatrics, SLK-Kliniken Heilbronn GmbH, Heilbronn, Germany

**Keywords:** Ependymoma, Recurrence, Child, Neoplasm metastasis, Magnetic resonance imaging

## Abstract

**Purpose:**

Currently, the different types of ependymal neoplasm (EPN) are defined by anatomical localization and genetics. This retrospective multicenter study aimed to analyze the imaging patterns of both local and distant recurrences in supratentorial (ST) and posterior fossa (PF) EPN.

**Methods:**

We exclusively evaluated patients with recurrent EPN. To form the basis for follow-up evaluations the imaging characteristics for ST-EPN and PF-EPN were assessed and compared to each other. Follow-up assessments included the idenTIFFication of local recurrent tumors, leptomeningeal dissemination, secondary intraparenchymal lesions, and extraneural metastases. MR-signal characteristics of local recurrent tumors were compared to the primary tumor.

**Results:**

The imaging series included 73 patients (median age at diagnosis 4.6 years; 56 PF-EPN). Recurrences were observed at up to five time points, with a total of 145 recurrence events documented. At first recurrence most PF-EPN recurred locally (29/56), while ST-EPN relapsed by intracranial dissemination (9/17). Local recurrent tumor grew fast and differed in up to one-fifth from the primary (13.2% lower T2-signal, 14.6% brighter T1-signal, 19% less contrast-enhancement). Leptomeningeal dissemination in ST-EPN is mainly restricted to intracranial (90.5%) while PF-EPN more frequently present with spinal spread (45.7%). Transient post-radiogenic lesions (*n* = 2) and secondary malignancies (*n* = 2) were rare. Extraneural metastases (*n* = 3) were found mainly near the surgical access.

**Conclusion:**

Recurrences can occur multiple times in EPN patients, and the recurrence patterns differ between ST-EPN and PF-EPN. Imaging characteristics of local recurrences can differ from the primary tumor which is crucial for accurate diagnosis and treatment planning.

## Introduction

Ependymal neoplasms (EPN) of the central nervous system (CNS) account for less than 10% of all malignant intracranial childhood tumors [1]. Approximately two-thirds of intracranial EPN cases manifest in the posterior fossa [2]. Supratentorial EPN (ST-EPN) typically occur at a mean age of 7.8 years and posterior fossa EPN (PF-EPN) at a mean age of 5 years, with a slightly higher incidence in boys [3].

The standard approach for treatment involves maximal safe resection followed by radiotherapy. However, recurrence poses a significant challenge across EPN subgroups, leading to poor survival rates among pediatric patients. A systematic review in a pediatric cohort of recurrent EPN reported a pooled median progression-free survival of 6.7 months and a pooled overall survival of 11.2 months, with supratentorial recurrences exhibiting shorter overall survival compared to infratentorial recurrences [4]. The HIT-REZ trials have recently indicated no significant impact of any kind of chemotherapy on recurrent EPN [5].

The histological differentiation between CNS-WHO grade 2 EPN and grade 3 anaplastic EPN has historically been challenging, and prognostic risk straTIFFication has been a subject of debate [6, 7]. Recently, molecular subtyping has come into focus. In 2015, nine distinct molecular ependymoma types were introduced [8, 9], which have been further refined in the fifth edition of the WHO classification [10, 11].

Among children, *ZFTA*-fusion positive ST-EPN (formerly termed ST-EPN with RELA-fusion) and PF-EPN group A (PF-A) constitute the majority of tumors at their respective anatomical sites. Clinical behavior varies between ST-EPN and PF-EPN subtypes. The ST-EPN subgroups, *ZFTA*-fusion and *YAP1*-fusion, are both highly recurrent but differ in outcomes, with *YAP1*-fusion tumors associated with a better prognosis [8]. Tumors in the PF-A group are associated with a significantly worse prognosis compared to PF-EPN group B tumors and all other subgroups [8, 12]. Post-relapse survival is poor for PF-A and *ZFTA*-fusion-positive EPN [13].

Imaging morphology of EPN at diagnosis is well-documented; however, most studies primarily compare genetic subgroups within a respective compartment or to other tumor entities in the same compartment. On magnetic resonance imaging (MRI), EPN exhibits heterogeneous contrast-enhancement and no or mild restriction on diffusion-weighted images (DWI) [14–16]. Despite single case reports and meta-analyses, limited information is available on the imaging morphology of recurrent EPN. A study by Massimino et al. indicated that EPN patients with symptomatic relapses carried a worse overall-survival compared to those with relapses detected on MRI without symptoms [17]. Thus, MRI plays a crucial role in the follow-up of EPN patients.

This study aims to offer a comprehensive description of pediatric ST-EPN compared to PF-EPN, along with a systematic evaluation of timing and imaging characteristics in local and distant recurrences.

## Methods

### Patients

Pediatric EPN patients with progressive disease following first-line treatment, evidenced by at least one new lesion on cranial and/or spinal follow-up MRI, were evaluated. Patients were retrospectively collected from the image database of recurrent tumors of the National Reference Center for Neuroradiology in January 2019. This retrospective evaluation was approved by the local institutional review board (No. 20231016 04) and was performed in accordance with the Declaration of Helsinki. Patients with recurrent EPN from multiple centers were included in the prospective HIT-REZ-2005 trial (NCT00749723) or registry after being initially included in the HIT 2000 [ trial (NCT00303810), I-HIT-MED Registry (NCT02417324) and HIT-Interim-Registry (NCT02238899) at diagnosis. Each patient or legal guardian provided informed consent before entering into the prospective brain tumor studies and registries.

### Pathology and molecular assessment

Neuropathological review was conducted by the [National Brain Tumor Reference Center of the German Society of Neuropathology and Neuroanatomy (DGNN) (Institute of Neuropathology, Bonn University, Germany). The molecular tumor type was determined using DNA methylation microarrays (Infinium HumanMethylation450 or EPIC BeadChip, Illumina, San Diego, California) at the German Cancer Research Center (DKFZ, University of Heidelberg, Germany). Data on molecular genetics were updated in 2023. However, since the patients were diagnosed starting in 2002, the determination of the molecular type was not available for every patient.

### Imaging assessment

In general, patients were examined according to the study protocols, which required cranial and spinal MRI to be performed before and after surgery, as well as every 2–4 months during the first-line treatment phase. In the first and second years after the end of therapy, cranial MRI was conducted every 3 months and spinal MRI every 6 months. During years 3 to 5 of follow-up, cranial MRI was performed every 6–9 months and spinal MRI every 6–12 months if a complete response had not been achieved. In years 6 to 10 after therapy, annual cranial MRI follow-ups were conducted, while spinal imaging was no longer mandatory. If clinical or radiological suspicion of recurrence arose, both cranial and spinal MRI were mandatory.

MRI datasets were assessed by two neuroradiologists dedicated to pediatric brain tumor imaging (M. W.-M. and A.S.). In cases of disagreement, a consensus reading was carried out. Inclusion criteria were a preoperative MRI at diagnosis and at least one follow-up MRI. For the local recurrent tumor assessment in this retrospective work-up, a regular postoperative MRI within 72 h was mandatory. In cases of incomplete tumor resection, only local recurrent tumors appearing distant from the residual primary tumor were assessed; progressive residual tumors were not evaluated to focus on radiological relapse patterns. If an early postoperative MRI was unavailable, only lesions outside the primary tumor site were considered for evaluation. The multicenter approach results in inconsistent MRI examinations in terms of field strength and sequencing technique, and limited information on treatment strategies.

#### Imaging at diagnosis

Primary tumor localization was categorized as infratentorial and supratentorial. Each anatomical site was further assessed for detailed localization: the solid part of ST-EPN for broad-based contact with the dura mater, ventricular wall, or both, or exclusively within the parenchyma; main tumor mass of PF-EPN for lateral localization within the cerebellopontine angle, lateral localization within the cerebellar hemisphere, midline localization within the fourth ventricle, midline localization within the foramen of Magendie, or midline localization with large lateral extension. Local standardized diagnostic parameters were supplemented and used for the imaging assessment as follows: The tumor volume was calculated using the approximation of the ellipsoid volume formula A x B x C x ½ where A, B, and C are the maximum dimensions in the standard anteroposterior, craniocaudal and transverse plane. Signal intensity on T2-weighed images (T2WI) and T1-weighted images (T1WI) without contrast were determined iso-, hyper- or hypointense compared to the supratentorial cortex. Homogeneity on T2WI and T1WI was assessed as homogeneous or inhomogeneous. Contrast intensity was rated as none, slight, moderate, or strong compared with usually strong contrast-enhancing tissue like mucosa. Amount of contrast enhancement can be diffuse in EPN and therefore, rating was subjective in approximate percentages of volume (≤ 25%; 26–50%; 51–75%; 76–100%). Hydrocephalus was rated as slight (only dilation of the ventricles), moderate (presence of periventricular pressure caps) or severe (compression of the sulci at the vertex). Tumor cysts and intratumoral necrosis were termed non-solid lesions, and when present, their intratumoral extension was rated in approximate percentages (more or less than 50% of tumor volume). Blood degradation products like met-hemoglobin or hemosiderin were evaluated for presence or absence on T1/T2WI or T2*/SWI. When DWI was available, the tumor was qualitatively assessed for restricted diffusivity based on B1000 images with correlation to signal reduction on the ADC map. The tumor’s diffusivity was compared to that of grey matter. Leptomeningeal dissemination was only determined on MRI and classified according to Chang [18]. Cranial dissemination was rated as M2, spinal dissemination as M3, and morphology was further rated as laminar (a) or nodular (b). When available, CT scans were assessed for intratumoral calcification and hemorrhage.

The extent of resection was radiologically determined as gross total (no macroscopic residual tumor), near gross total (measurable residual tumor, maximum diameter ≤ 0.5 cm), subtotal (measurable residual tumor maximum diameter ≥ 0.5 cm) and debulking/biopsy.

#### Imaging at recurrence

New expansive lesions observed at the site of the primary tumor and along the resection cavity were considered local recurrent tumors. MRIs of the primary tumor and the first local recurrent tumor (time-point 1) were assessed for signal intensity, homogeneity, and contrast-enhancement on T1WI and T2WI. Ratings were assigned as ‘more,’ ‘less,’ or ‘stable’ based on a comparison with the initial MRI. Local recurrent tumor volume and perifocal edema were measured as on the initial MRI. In patients with multiple local recurrent tumors on follow-up, only the first local recurrent tumor present was compared to the primary tumor. Furthermore, the first local recurrent tumor was evaluated on follow-up before re-resection (time-point 2) when available, and was compared to time-point 1 and to the primary tumor.

New lesions within the brain parenchyma on T2WI and post-contrast T1WI outside the primary tumor site were classified as secondary lesions. Secondary lesions after radiotherapy that regressed without specific treatment on follow-up were rated as transient post-radiogenic lesions.

New cranial and spinal leptomeningeal lesions were assessed as leptomeningeal dissemination. Initial dissemination was not evaluated on follow-up. M3 was only assessed for presence. M2b was evaluated for contrast-enhancement and restricted diffusivity on DWI when available. When M2b’s parameters, signal intensity contrast-enhancement, and non-solid lesions were comparable to the primary tumor, this was documented as well.

New lesions outside the CNS, within in the field-of-view of the cranial or spinal MRI, were deemed extraneural metastases.

### Statistics

Data analysis was performed with SPSS Statistics (IBM Corp. Released 2021. IBM SPSS Statistics for Windows, Version 28.0. Armonk, NY: IBM Corp). A group comparison was conducted between ST-EPN and PF-EPN, as complete molecular genetic information was not available and because *YAP1*-fusion-positive and PF-B EPN are particularly rare. Continuous variables like age and tumor volume are described by median and range. Medians between ST-EPN and PF-EPN were compared utilizing Mann-Whitney-U test. For categorical variables (sex, degree of resection, type of recurrence and imaging parameters both at diagnosis and in relapse), absolute and relative frequencies are given. Comparison between ST-EPN and PF-EPN with categorical variables was performed with Fisher exact test because of small group size. A *p* value ≤ 0.05 was considered as meaningful effect.

## Results

### Patients

A total of 107 patients with recurrent EPN were retrospectively collected. Of the total cohort, 34 were excluded because of incomplete MR scans (*n* = 26), only progressive residual tumor (*n* = 7) or adult age at diagnosis (*n* = 1). The final cohort consisted of 73 patients (median age at diagnosis 4.6 years; range 0.3-15.1years; 57.5% boys). Each patient experienced at least one recurrence, with up to five recurrence time points identiffied in some cases. In total, 145 radiological recurrences of various types were documented.

### Pathology and molecular assessment

Among the 73 patients, 56 (76.7%) were PF-EPN with confirmation of PF-A in 41 and PF-B in 1 (age at diagnosis 7.3 years). Molecular subgroup information was not available for 14 PF-EPN patients. For 14 out of 17 ST-EPN, molecular data were available, with 13 harboring a *ZFTA*-fusion and one a *YAP1*-fusion (age at diagnosis 0.4 years). Histopathology revealed anaplastic EPN in all patients except for one case of PF-EPN with WHO grade 2.

### Imaging assessment

#### Imaging at diagnosis

##### Localization

ST-EPN showed in 6/17 broad-based contact to the dura mater, in 4/17 broad-based contact to the ventricular wall, four showed both and three tumors were solely intraparenchymal distanced to the dura mater and the ventricular wall. PF-EPN were located within the midline in more than two-thirds (41/56; *n* = 17 within the fourth ventricle, *n* = 11 in the Foramen of Magendie, *n* = 13 midline localization with large lateral extension into the cerebellopontine angle). One PF-EPN was radiologically localized in the cerebellar hemisphere, and 14 in the cerebellopontine angle.

##### Imaging characteristics

**Fig. 1:**
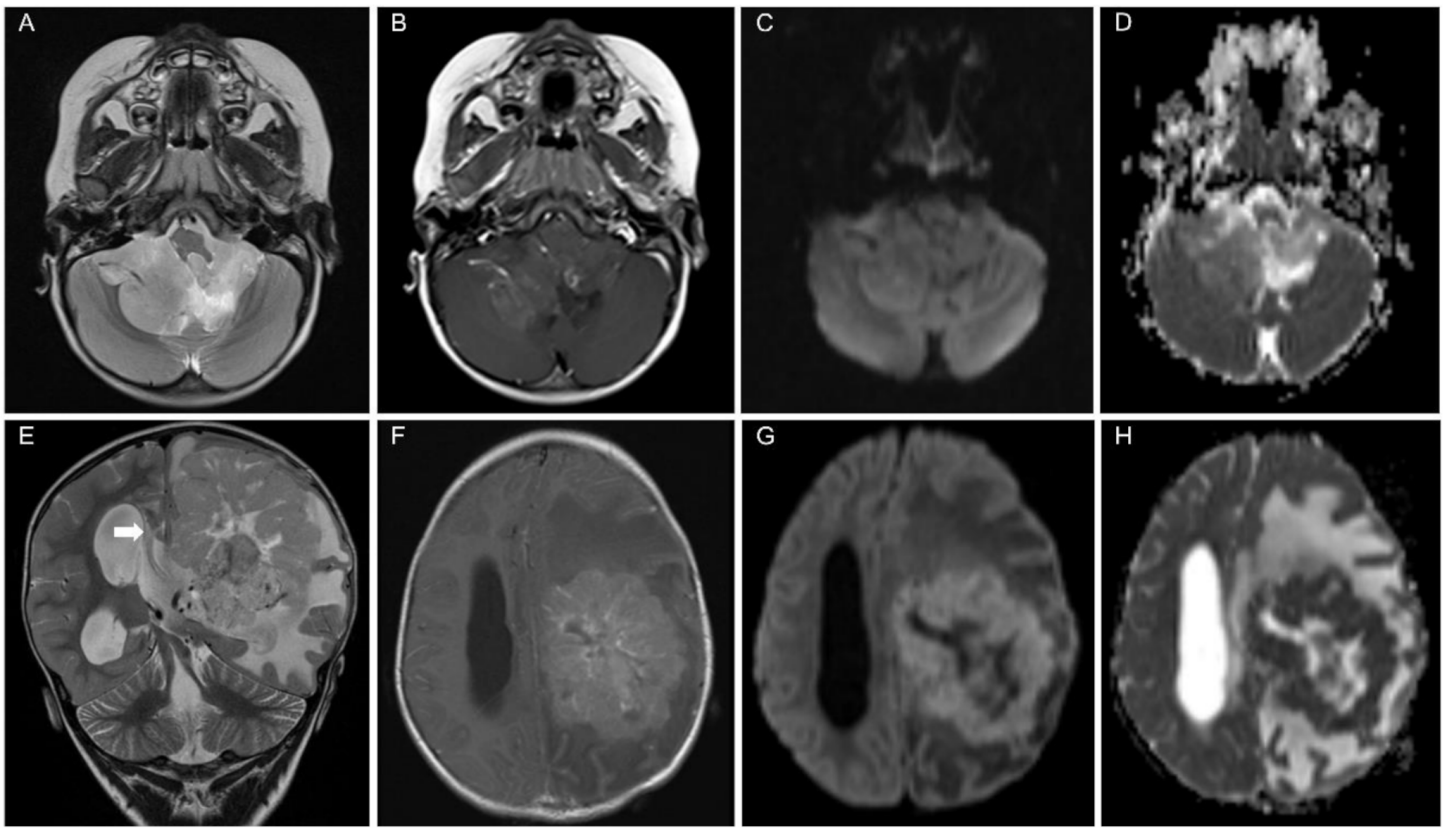
A 2-year-old girl with a posterior fossa ependymoma (**A**-**D**) and a 2-year-old boy with a supratentorial ependymoma (**E**-**H**), both at diagnosis. (**A**-**D**) The upper row shows the typical appearance of a posterior fossa ependymoma. (**A**) The signal on T2-weighted images is brighter than the cortex. (**B**) Only a portion of the tumor shows moderate contrast-enhancement. (**C**) On B1000 DWI image, the tumor shows intermediate signal without unequivocal restriction since the signal on ADC map (**D**) is predominately brighter than the cortex. (**E**-**H**) The lower row presents a *ZFTA*-fusion positive supratentorial ependymoma. (**E**) The large tumor mass with massive edema and moderate contrast-enhancement (**F**) in the left hemisphere led to subfalcine herniation (white arrow in **E**) and hydrocephalus. Restriction on diffusion-weighted images (B1000 image in **G** and corresponding ADC map in **H**) and intermediate signal intensity on T2-weighted images (**E**) illustrate high cell density on MRI, which is noticeably in contrast to the posterior fossa ependymoma

ST-EPN had a larger median volume compared to PF-EPN (*p* < 0.001). Perifocal edema was more prevalent in ST-EPN. Hydrocephalus was less frequent and less severe in ST-EPN than in PF-EPN (*p* < 0.001). Imaging characteristics varied (Fig. 1; Table 1), with PF-EPN exhibiting brighter T2WI (*p* = 0.015) and lower T1WI (*p* = 0.009) signal intensity than ST-EPN. Restricted diffusivity was significantly more common in ST-EPN (*p* < 0.001). Contrast-enhancement was observed in all tumors, with non-solid lesions notably larger in ST-EPN than PF-EPN (*p* = 0.017). There was no difference in T1WI or T2WI homogeneity or in contrast-enhancement behavior. Approximately half of ST-EPN (52.9%) and PF-EPN (64.3%) showed intratumoral iron-sensitive components on MRI. In two patients, M3 disease was found (one ST-EPN, one PF-EPN). No patient presented with M2 stage at diagnosis. Pre-surgical non-contrast CT (*n* = 7) showed intratumoral calcifications in each tumor but only two PF-EPNs showed hemorrhages. Surgical resection was more extensive in ST-EPN (86.7% gross total resection versus 59.3% in PF-EPN).

**Table 1:** Imaging analyses of the primary tumor

	total cohort	ST-EPN		PF-EPN		*P* value
	*n* = 73	total *n* = 17^1^	*ZFTA*-fusion positive (*n* = 13)	total *n* = 56^2^	PF-A (*n* = 41)	
median age at diagnosis (in years)	4.6 (0.3–15.1)	4.3 (0.3–14.6)	4.4 (1.5–12.5)	4.6 (0.4–15.1)	4.6 (0.4–15.1)	0.59
gender						0.4
• male	42 (57.5%)	8 (47.1%)	7 (53.8%)	34 (60.7%)	24 (58.5%)	
• female	31 (42.5%)	9 (52.9%)	6 (46.3%)	22 (39.3%)	17 (41.5%)	
**Imaging at diagnosis**						
median tumor volume (in cm^3^)	38.2 (3.7-303.4)	114.2 (3.7-303.4)	111.2 (3.7-303.4)	31.8 (9.3-147.4)	30.1 (9.3-147.4)	< 0.001
hydrocephalus	*n* = 73					< 0.001
• none	18 (24.7%)	11 (64.7%)	8 (61.5%)	7 (12.5%)	5 (12.2%)	
• mild	11 (15.1%)	0	0	11 (19.6%)	8 (19.5%)	
• moderate	34 (46.6%)	4 (23.5%)	3 (23.1%)	30 (53.6%)	21 (51.2%)	
• severe	10 (13.7%)	2 (11.8%)	2 (15.4%)	8 (14.3%)	7 (17.1%)	
non-solid lesions	*n* = 73					
• none	6 (8.2%)	0	0	6 (10.7%)	5 (12.2%)	
• ≤ 50%	42 (57.5%)	6 (35.3%)	5 (38.5%)	36 (64.3%)	27 (65.9%)	0.017
• > 50%	25 (34.2%)	11 (64.7%)	8 (61.5%)	14 (25%)	9 (22%)	
T1WI	*n* = 72	*n* = 17	*n* = 13	*n* = 55	*n* = 41	
I. homogeneity						1
• homogeneous	54 (74%)	13 (76.5%)	10 (76.9%)	41 (74.5%)	32 (78%)	
• inhomogeneous	18 (24.7%)	4 (23.5%)	3 (23.1%)	14 (25.5%)	9 (22%)	
II. signal-intensity						0.009
• hyperintense	1 (1.4%)	1 (5.9%)	1 (7.7%)	0	0	
• isointense	57 (78.1%)	16 (94.1%)	12 (92.2%)	41 (74.5%)	32 (78%)	
• hypointense	14 (19.2%)	0	0	14 (25.5%)	9 (22%)	
T2WI	*n* = 71	*n* = 17	*n* = 13	*n* = 54	*n* = 39	
I. homogeneity						1
• homogeneous	24 (32.9%)	6 (35.3%)	3 (23.1%)	18 (33.3%)	12 (30.8%)	
• inhomogeneous	47 (64.4%)	11 (64.7%)	10 (76.9%)	36 (66.7%)	27 (69.2%)	
II. signal intensity						0.015
• hyperintense	48 (65.8%)	7 (41.2%)	6 (46.2%)	41 (75.9%)	29 (74.4%)	
• isointense	23 (31.5%)	10 (58.8%)	7 (53.8%)	13 (24.1%)	10 (25.6%)	
• hypointense	0	0	0	0	0	
contrast intensity	*n* = 73					0.65
• none	0	0	0	0	0	
• slight	9 (12.3%)	2 (11.8%)	1 (7.7%)	7 (12.5%)	5 (12.2%)	
• moderate	15 (20.5%)	2 (11.8%)	1 (7.7%)	13 (23.2%)	9 (22%)	
• strong	49 (67.1%)	13 (76.5%)	11 (84.6%)	36 (64.3%)	27 (65.9%)	
amount of enhancement	*n* = 73					0.2
• ≤ 25%	2 (2.7%)	0	0	2 (3.6%)	1 (2.4%)	
• ≤ 50%	6 (8.2%)	0	0	6 (10.7%)	5 (12.2%)	
• ≤ 75%	17 (23.3%)	2 (11.8%)	1 (7.7%)	15 (26.8%)	10 (24.4%)	
• > 75%	48 (65.8%)	15 (88.2%)	12 (92.3%)	33 (58.9%)	25 (60.1%)	
blood degradation products	*n* = 73					0.4
• present	45 (61.6%)	9 (52.9)	6 (46.2%)	36 (64.3)	24 (58.5%)	
• absent	28 (38.4)	8 (47.1)	7 (53.8%)	20 (35.7)	17 (41.5%)	
restricted diffusion	*n* = 47	*n* = 14	*n* = 11	*n* = 33	*n* = 23	< 0.001
• yes	17 (36.2%)	13 (92.9%)	10 (90.9%)	4 (12.1%)	0	
• no	30 (63.8%)	1 (7.1%)	1 (9.1%)	29 (87.9%)	23 (100%)	
dissemination	2 (2.7%)	1 (5.9%)	1 (7.7%)	1 (1.8%)	1 (2.4%)	0.4
**pre-operative CT**	total *n* = 7	*n* = 1	*n* = 1	*n* = 6	*n* = 4	1
• calcifications	5 (71.4%)	1 (100%)	1 (100%)	4 (66.7%)	3 (75%)	
• hemorrhage	1 (14.3%)	0	0	1 (16.7%)	0	
• both	1 (14.3%)	0	0	1 (16.7%)	1 (25%)	
**initial degree of resection** ^**3**^	total *n* = 69	*n* = 15	*n* = 11	*n* = 54	*n* = 40	0.3
- gross total	45 (65.2%)	13 (86.7%)	9 (81.8%)	32 (59.3%)	27 (67.5%)	
- near gross total	7 (10.1%)	1 (6.7%)	1 (9.1%)	6 (11.1%)	4 (10%)	
- subtotal	15 (21.7%)	1 (6.7%)	1 (9.1%)	14 (25.9%)	8 (20%)	
- debulking or biopsy	2 (2.9%)	0	0	2 (3.7%)	1 (2.5%)	

Table 1 shows the absolute and relative frequencies of patient data and parameters from CT and MRI scans at diagnosis for the total cohort. It is further divided by anatomical site (ST-EPN and PF-EPN) and molecular subgroup. Please note that the molecular subgroup information was not available for all patients (3 ST-EPN and 14 PF-EPN). At diagnosis, one patient had no evaluable T1WI pre contrast and two patients had no evaluable T2WI. The *P* values are given for a group comparison between ST-EPN and PF-EPN. The group comparison for blood degradation products was only analyzed for presence or absence regardless of the sequence technique used. Abbreviations: CT = computed tomography; PF = posterior fossa; PF-A = posterior fossa group A; PF-EPN = posterior fossa ependymoma; ST = supratentorial; ST-EPN = supratentorial ependymoma; T1WI = T1-weighted images; T2WI = T2-weighted images

^1^ Including one *YAP1*-fusion positive tumor

^2^ Including one posterior fossa group B tumor

^3^ Early postoperative MRI was not available in four patients (2 ST-EPN, 2 *ZFTA*-fusion positive EPN; 2-PF-EPN, 1 PF-A)

#### Imaging at recurrence

##### Recurrence in general

Every patient experienced at least one recurrence event, with up to five time-points of new lesions observed in available MR scans (Table 2). The median time to first recurrence for the entire cohort was 1.6 years, with ST-EPN at 1.2 (0.2–4.8) years and PF-EPN at 1.6 (0.08–8.7) years. PF-EPN primarily recurred locally (29/56), followed by M3 (12/56) and M2 (9/56). A first recurrence by local recurrent tumor and simultaneous M2 or M3 occurred in four PF-EPN patients. ST-EPN recurred more often with M2 (9/17) and locally (7/17), but not with M3. A secondary lesion was the first relapse in one ST-EPN (1/17) and in two PF-EPN (2/56) patients. The modes of relapse significantly differed between supra- and infratentorial localization (*p* = 0.022).

**Table 2:** Frequency and types of recurrences

type of recurrence	1st recurrence	2nd recurrence	3rd recurrence	4th recurrence	5th recurrence
local recurrent tumor	36 (49.3%)	20 (43.5%)	7 (41.2%)	1 (14.3%)	0
1st	*40* *	*5*	*-*	*-*	*-*
2nd	*-*	*16*	*2*	*-*	*-*
3rd	*-*	*-*	*6*	*-*	*-*
4th	*-*	*-*	*-*	*2*	*-*
M2	18 (24.7%)	15 (32.6%)	7 (41.2%)	2 (28.6%)	2 (100%)
M3	12 (16.4%)	8 (17.4%)	2 (11.8%)	1 (14.3%)	0
local recurrent tumor + M2	2 (2.7%)	0	1 (5.9%)	1 (14.3%)	0
recurrent tumor + M3	2 (2.7%)	1 (2.2%)	0	0	0
secondary lesion	3 (4.1%)	1 (2.2%)	0	0	0
extraneural metastasis	0	1 (2.2%)	0	1 (14.3%)	0
extraneural metastasis + M2	0	0	0	1 (14.3%)	0
total	73	46	17	7	2

Table 2 shows the frequency of recurrences per patient and the type of recurrence. Additional data are provided for locally recurrent tumors because local recurrences sometimes occurred after other types of recurrences. Abbreviations: M2 = macroscopic intracranial metastasis; M3 = macroscopic intraspinal metastasis

* At the time the local recurrent tumor was diagnosed, there were two patients with additional M2 and two patients with additional M3 (*n* = 4 combined relapses)

##### Imaging of local recurrences at time-point 1

A total of 45 patients exhibited local recurrent tumors, with 38 primarily PF-EPN and 7 ST-EPN. The median time between surgery and first local recurrent tumor was 1.7 years. The median volume of local recurrent tumors was 0.2 cm³ (range 0.004–11.9 cm³), with ST-EPN (median 1.4 cm³, range 0.2–11.9) having larger local recurrent tumors than PF-EPN (median 0.15 cm³, range 0.004-8.2; *p* = 0.003). Imaging characteristics of local recurrent tumors were generally comparable to the primary tumor (Table 3), with some signal intensity variations. Lower amount of contrast-enhancement was present in 19.1%, a lower T2-signal in 13.2%, a brighter T1-signal in 14.6%, and non-solid intratumoral lesions were less frequent (59.5%).

**Table 3:** Comparison of MR imaging characteristics between local recurrent ependymoma and primary tumors

	time-point 1 to primary tumor	time-point 2 to time-point 1	time-point 2 to primary tumor
**T2-signal**			
**I. intensity** - similar - more intense - less intense	n = 38* 33 (86.8%) - 5 (13.2%)	n = 21* 19 (90.5%) - 2 (9.5%)	n = 22* 19 (86.4%) 1 (4.5%) 2 (9.1%)
**II. homogeneity** - similar - more homogeneous - less homogeneous	n = 38* 30 (78.9%) 7 (18.4%) 1 (2.6%)	n = 21* 16 (76.2%) - 5 (23.5%)	n = 22* 15 (68.2%) 4 (18.2%) 3 (13.6%)
**T1-signal (without contrast)**			
**I. intensity** - similar - more intense - less intense	n = 41* 33 (80.5%) 6 (14.6%) 2 (4.9%)	n = 23* 21 (91.3%) - 2 (8.7%)	n = 24* 20 (83.3%) 3 (12.5%) 1 (4.2%)
**II. homogeneity** - similar - more homogeneous - less homogeneous	n = 41* 35 (85.4%) 5 (12.2%) 1 (2.4%)	n = 23* 22 (95.7%) - 1 (4.3%)	n = 24* 16 (66.7%) 7 (29.2%) 1 (4.2%)
**contrast-enhancement**			
**I. intensity** - similar - stronger - less	n = 42* 32 (76.2%) 3 (7.1%) 7 (16.7%)	**n = 29** 18 (62.1%) 11 (37.9%) -	**n = 29** 23 (79.3%) 3 (10.3%) 3 (10.3%)
**II. volume** - similar - more - less	n = 42* 29 (69%) 5 (11.9%) 8 (19.1%)	n = 26* 17 (65.4%) 9 (34.6%) -	n = 27* 14 (51.9%) 9 (33.3%) 4 (14.8%)
**blood degradation products** - similar - more - less	n = 39* 22 (56.4%) - 17 (43.6%)	n = 22* 19 (86.4%) 3 (13.6%) -	n = 21* 11 (52.4%) 2 (9.5%) 8 (38.1%)
**non-solid intratumoral lesions** - similar - more - less	n = 42* 14 (33.3%) 3 (7.1%) 25 (59.5%)	**n = 29** 14 (48.3%) 15 (51.7%) -	**n = 29** 9 (31%) 4 (13.8%) 16 (55.2%)
**restricted diffusivity** - similar - more - less	n = 19* 15 (78.9%) - 4 (21.1%)	n = 8* 6 (75%) 1 (12.5%) 1 (12.5%)	n = 9* 5 (55.6%) 2 (22.2%) 2 (22.2%)

This table compares the MR imaging characteristics between the first local recurrent tumor at diagnosis (time-point 1; total *n* = 45) and the primary tumor. Additionally, the first local recurrent tumor was evaluated again (time-point 2; total *n* = 29) when a further MRI pre re-resection was available. The time-point 2 MRI was compared to both the primary tumor and the time-point 1 MRI. Note that some local recurrences were too small for precise comparison at time-point 1. Furthermore, in some cases, the sequence on at least one MRI was not available for comparison (marked with *). *n* = number of sequences available for comparison

It was also examined whether there was a distinction between the characteristics of local recurrent ST-EPN and PF-EPN at time-point 1. However, changes in imaging characteristics were not dependent on the site of the primary tumor (T2-signal *p* = 1, T2 homogeneity *p* = 1, T1-signal *p* = 0.51, T1 homogeneity *p* = 0.64, contrast-enhancement *p* = 0.45, amount of contrast-enhancement *p* = 0.28, non-solid lesions *p* = 0.08, blood degradation products *p* = 0.11, and restricted diffusivity *p* = 0.097).

##### Local recurrences at time-point 2

Of 45 patients with a first local recurrent tumor, 29 had additional pre-surgical MRIs (time-point 2) a median of 112 (interquartile range 47–294) days later.

Five of the 29 patients have continued with first-line chemo- or radiotherapy due to early tumor progression, and 9 patients with relapses following first-line therapy were treated with interim chemo- or radiotherapy (including one iodine-seed implantation). However, the other 15 patients did not receive any therapy between time-point 1 and time-point 2. Of these 15 patients, a tumor biopsy only was performed without tissue reduction in one patient and the local recurrence was totally resected a few months later. Clinical reports revealed that relapse was detected in 25 out of 29 patients (12 at time-point 1, but two were interpreted as post-therapeutic changes; five at time-point 2; and eight in the meantime). Relapse was not detected in two cases, and no information was available for two other cases.

##### Imaging of local recurrences at time-point 2

**Fig. 2:**
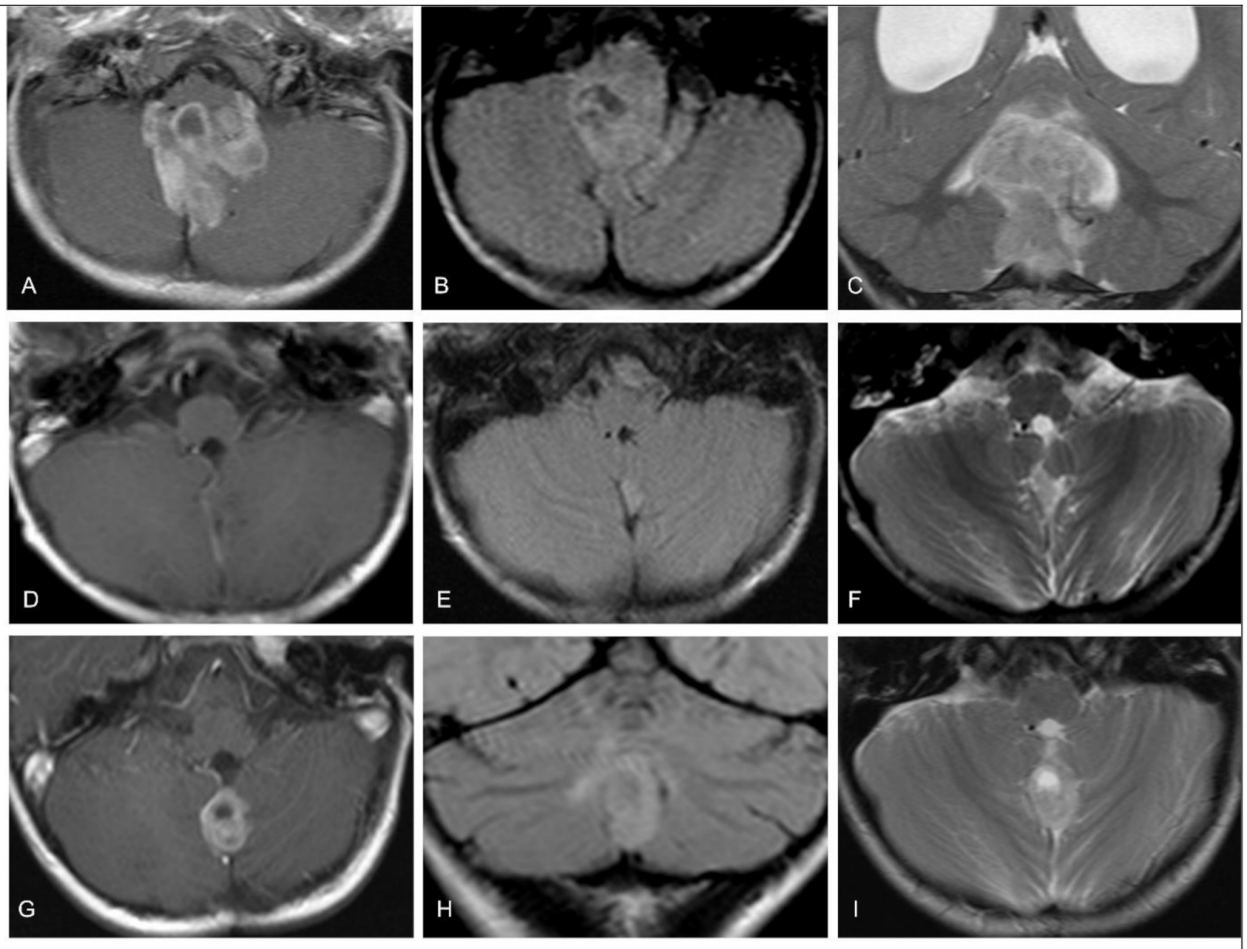
A 1-year-old girl with a posterior fossa ependymoma. (**A**-**C**) At diagnosis. The primary tumor showed strong contrast-enhancement (**A**) and inhomogeneous hyperintense signal intensity on T2-weighted and FLAIR images (**B**, **C**). (**D**-**F**) Local recurrent tumor at time-point 1, two years after total resection. The new lesion is best seen on T2-FLAIR (**E**) and T2 (**F**). T2-signal was similar compared to the primary tumor but there was lack of contrast-enhancement (**D**). (**G**-**I**) Local recurrent tumor at time-point 2, 22 months after time-point 1. The recurrent tumor now shows signal intensities resembling the primary tumor

Median tumor volume at the second local recurrent tumor time-point was 0.7 cm³ (range 0.01–7.83 cm³). The tumor with the shortest distance between time-point 1 and 2 gained 67% in volume within 14 days (time-point 1: 1.2 cm³; time-point 2: 2 cm³). Changes in imaging parameters were observed aligning more with the primary tumor, with contrast-enhancement increasing in 11/29 patients. Eight of the 11 patients were undergoing treatment, including five who were receiving radiotherapy. In two of the 18 cases with stable contrast-enhancement intensity, the amount of contrast-enhancement increased; in both instances, no therapy was administered during that period. T1WI and T2WI differences were less pronounced at time-point 2 compared to the primary tumor (Fig. 2). Of the 45 local recurrent tumors, biopsy or resection was performed in 36 patients, with no changes in WHO grade. PF-A was idenTIFFied in three patients with initial anaplastic PF-EPN without genetic information.

##### Leptomeningeal dissemination

**Fig. 3:**
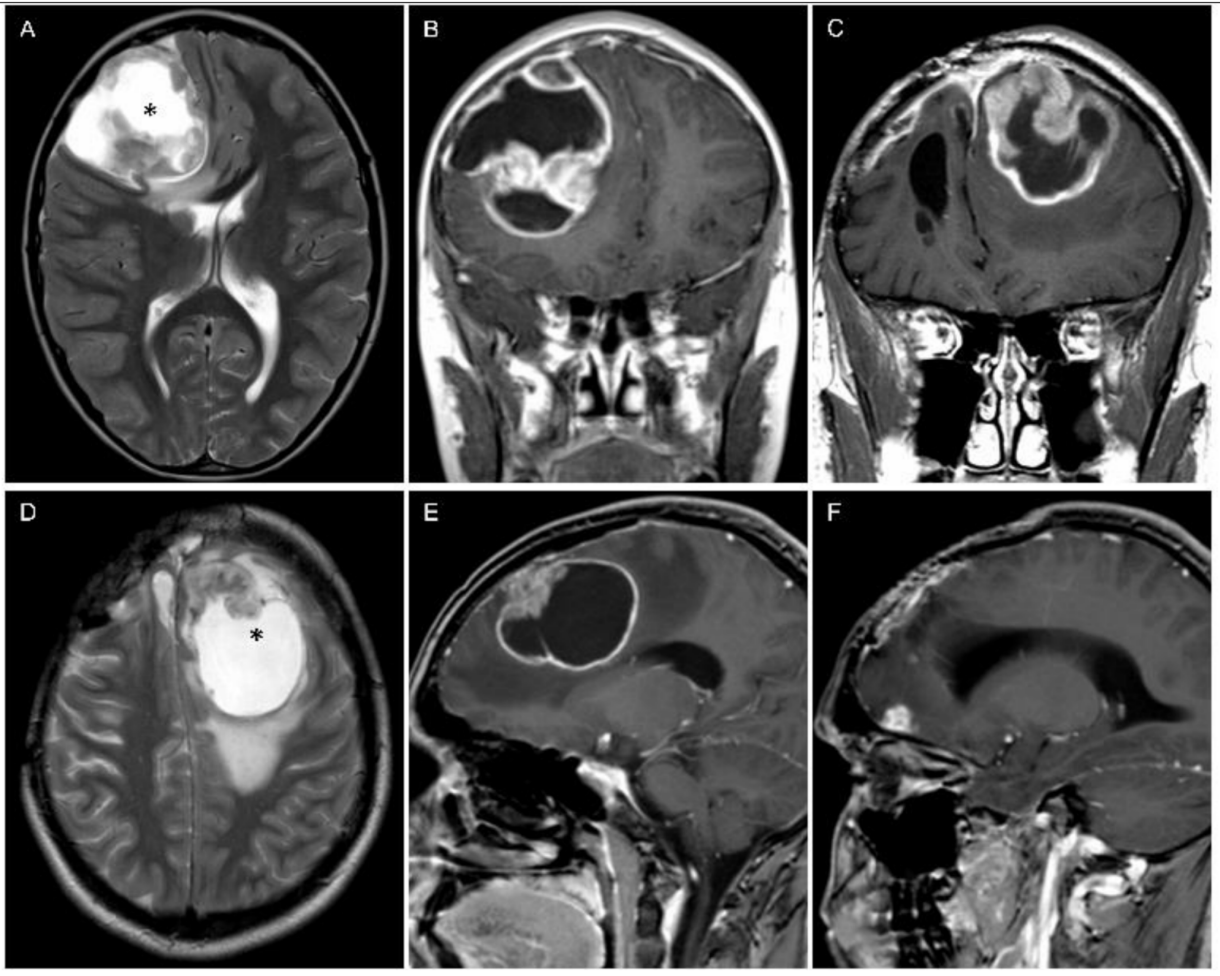
A 9-year-old boy with a *ZFTA*-fusion positive supratentorial ependymoma in the right frontal lobe at diagnosis on T2-weighted (**A**) and post-contrast T1-weighted images (**B**). The patient had at first relapse a local recurrent tumor 43 months after initial total resection. On follow-up two further local recurrences occurred. The local recurrent tumors never crossed the midline. (**C**-**E**) The fourth recurrence was a left sided leptomeningeal metastasis with very similar signal intensities on T2-weighted (**D**) and post-contrast T1-weighted images (**C**, **E**). The tumor tissue contained a large central non-solid area (*) at diagnosis (**A**) and in relapse (**D**). (**E**) shows the broad-based contact of the metastasis to the dura mater. (**F**) The patient simultaneously showed a second leptomeningeal metastasis at the right frontal base

On follow-up, recurrence was caused by M3 in 26 cases and in 49 cases by M2, in 8 with concomitant local or extraneural recurrences. When exclusively considering cases with either M2 or M3 involvement, a significant difference between ST-EPN and PF-EPN becomes evident. M3 was considerably more frequent in PF-EPN across all recurrence time points (21/46; 45.7%). In contrast, the frequency of M2 was significantly higher in ST-EPN (19/21; 90.5%; *p* = 0.005). Since cranial MRIs, unlike spinal MRIs, frequently included DWI sequences, M2 imaging morphology could be described in greater detail. All M2 were nodular, only in one patient M2a was present simultaneously (Table 4). The majority showed M2b with contrast-enhancement (38/49) and restricted diffusion (25/38). Although the M2 of ST-EPN more frequently exhibited diffusion restriction (80%) than that of PF-EPN (56.5%), the difference was not significant (*p* = 0.14). Restricted diffusion without contrast-enhancement was rare (3/38). Of the 49 M2b in 41 patients 18 displayed similar imaging characteristics compared to the primary tumor (Fig. 3).

**Table 4:** Appearance of nodular leptomeningeal intracranial metastases (M2b) on MRI in recurrence

MR imaging characteristics of M2b	total *n* = 49 in 41 patients
+ CE*	8
- CE*	3
+ CE + restricted diffusion	22**
+ CE - restricted diffusion	8
- CE + restricted diffusion	3
- CE - restricted diffusion	5

Abbreviations: (+) = present; (-) = absent; CE = contrast-enhancement; DWI = diffusion-weighted images

* DWI was not available in 11 patients. ** Including one patient with simultaneous laminar leptomeningeal dissemination (M2a)

##### Extraneural metastases

**Fig. 4:**
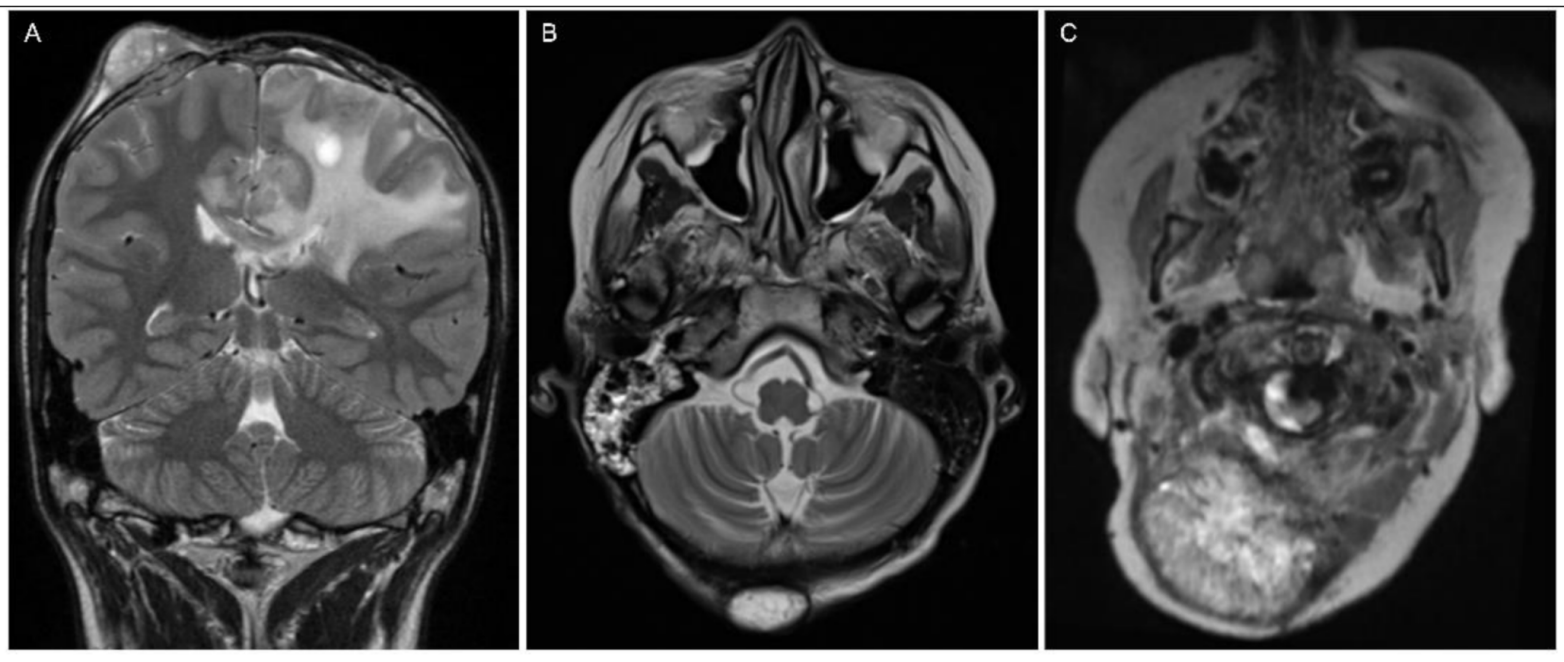
Extraneural metastases on follow-up (T2-weighted images). (**A**) A male patient diagnosed with a supratentorial ependymoma at the age of 10 developed a new subcutaneous soft tissue lesion near the first surgical access more than four years after the first resection. (**B**) A female patient diagnosed with a supratentorial ependymoma at the age of 11 developed a new subcutaneous lesion without connection to the surgical access more than four years after diagnosis. Here, no lymph nodes were present on earlier MRI studies. (**C**) A male patient diagnosed with a posterior fossa ependymoma at the age of 4 developed a large extracranial mass along the way of surgical access six years later

Extracranial metastases were observed in three patients (Fig. 4). In one patient, 32 months after recurrence by M2b, an extracranial metastasis developed in the occipital subcutis. In two patients at fourth recurrence, the metastases were located subcutaneously upon the frontal and suboccipital skull adjacent to the operative osseous defect. There was no histopathological confirmation of the metastases.

##### Secondary lesions

**Fig. 5:**
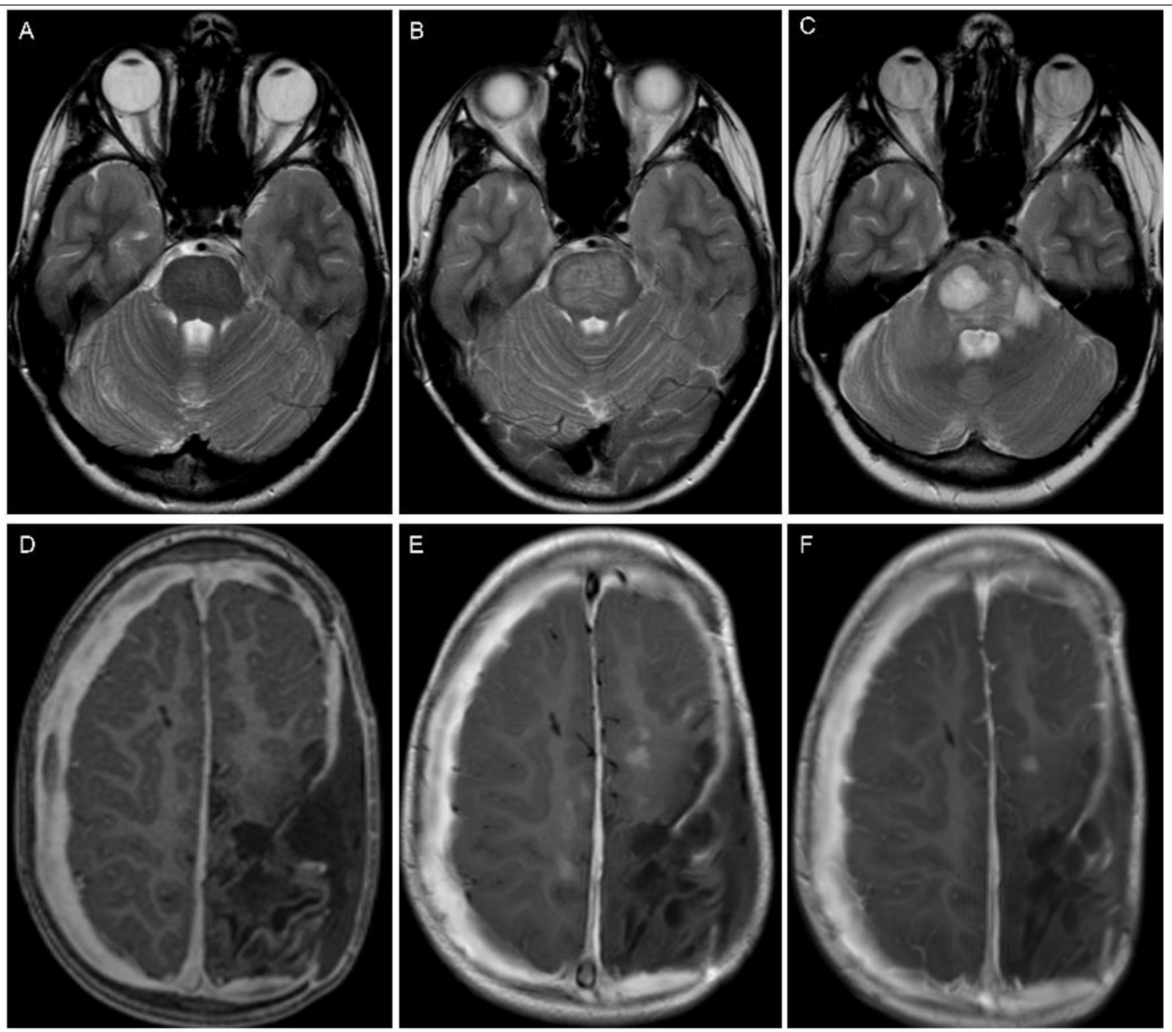
Examples of secondary lesions. (**A**-**C**) A 12-year-old boy diagnosed with a posterior fossa ependymoma developed a diffuse pontine lesion 23 months after diagnosis and a diffuse intrinsic pontine glioma was suspected. (**A**) Tumor manifestation started with a slight and diffuse increase in T2-signal intensity within the central pons. (**B**) MRI 10 months later shows the growth of the pontine T2-lesion, now fulfilling the criteria for a diffuse pontine glioma. (**C**) On further follow-up, the tumor is expanding into the cerebellar hemisphere. (**D**-**F**) A 2-year-old boy diagnosed with a supratentorial ependymoma (MRI at diagnosis in Fig. 1). (**D**) The first MRI control six weeks after proton therapy shows the left sided postoperative parenchymal defect without tumor residues but bilateral subdural hygroma on T1-weighted post-contrast images. (**E**) Six months after radiotherapy, new contrast-enhancing spots occur nearby the resection cavity, but also within primarily healthy brain tissue in the area of the right-sided central region. (**F**) Follow-up three months later shows regression of the contrast-enhancing spots, which supported the assumption of transient post-radiogenic imaging changes

Secondary lesions appeared in four patients (median survival 4.5 years; two examples in Fig. 5). Less than 2 years after diagnosis, one patient with primary PF-EPN developed a new pontine lesion, which was suspected to be a diffuse intrinsic pontine glioma based on imaging criteria. Further, one PF-EPN patient developed a new central pontine lesion 45 months after diagnosis, also fulfilling the criteria for a diffuse intrinsic pontine glioma. Histological analysis confirmed an astrocytoma, WHO grade 3, supporting the suspected diagnosis. However, unfortunately molecular genetic testing was not available at that time to confirm the presence of an H3 K27 mutation.

The one PF-B patient showed 16 months after diagnosis and 13 months after hyperfractionated radiotherapy with 68 Gy, a new parenchymal lesion within the right cerebellar peduncle within the field of radiation. The lesion was regressive after 4 months and therefore rated as a radiation induced lesion. A fourth patient showed multiple contrast-enhancing spots within the supratentorial brain adjacent to the primary tumor localization but as well on the contralateral hemisphere 5 months after proton beam therapy. Due to spontaneous regression after 2 months and a comparison of the MRI with the radiation plan, the changes could be classified as a transient post-radiogenic phenomenon.

## Discussion

This study systematically compared MRI characteristics of primary tumors by site and investigated various types of local and distant recurrences, as well as new lesions in children with intracranial EPN, all treated within the HIT-REZ-study. This study run by the German Society for Pediatric Oncology and Hematology (GPOH) and is taking care of recurrent CNS tumors of various histologies. The oncological aspects of the HIT-REZ EPN cohort, including also spinal EPN, were published in 2021 [19].

### Imaging at diagnosis

Previously, ST-EPNs have been described as generally periventricular tumors [14]. Nevertheless, the WHO classification has already reported the occurrence of ST-EPN outside the ventricular system and in some publications the localization was described as superficial with pial and cortical involvement [10, 20, 21]. A recent report idenTIFFied all ependymomas as intra-axial tumors [22]. In our cohort, ST-EPN exhibited mainly large volumes, hindering the assessment of their point of origin. As more than one-third (35.3%) of ST-EPN show a broad-based contact with the dura mater without reaching the ventricular wall, we can support the notion that ST-EPN are not exclusively periventricular tumors. Imaging in intracranial EPN, classified by anatomical site and molecular type, have been evaluated in detail. Nowak et al. [23] described the MRI phenotype of *RELA*-fusion positive EPN, revealing a 100% restricted diffusivity in those tumors, which is consistent with our observation. Leclerc et al. [24] compared PF-A and PF-B subgroups, discovering that PF-EPN is hyperintense on T2WI, comparable with our findings.

A direct comparison of the MRI characteristic between ST-EPN and PF-EPN is rare [25]. When comparing only the primary tumor sites, we found substantial differences in signal intensity on T1WI and T2WI. ST-EPNs appeared brighter in T1WI and darker in T2WI than PF-EPN. The T1WI and T2WI homogeneity was not specific. Furthermore, the most concise difference was found on DWI. Our results are in line with Kuai et al. [25]. ST-EPNs show restricted diffusivity, suggesting a higher cell density than PF-EPNs. Leptomeningeal dissemination at diagnosis was rare as previously described [26, 27].

### Imaging at recurrence

Relapse occurred up to five times which is in line with recently published reports [28]. Local recurrences were the main cause of the first progression after initial surgery, followed by metastatic recurrences (M2 and/or M3). PF-EPN, which were mainly PF-A, recurred most often locally, but this number is smaller than the reported local relapse in a larger multicentric cohort of PF-A presenting nearly two-third at the initial tumor bed [13]. The local recurrent tumors were small and, therefore, challenging to idenTIFFy or interpret. Since gross-total resection of local recurrent tumor at first relapse is associated with an improved survival, local recurrent tumor idenTIFFication at small stages is essential [29]. Local recurrent tumor and primary tumor differed in the following parameters: diminished contrast-enhancement was present in 19.1%, a lower T2-signal in 13.2%, a brighter T1-signal in 14.6%. Differences of presence or absence of blood degradation products and non-solid lesions compared to the primary tumor may be explained by the tiny sizes of local recurrent tumors. The first local recurrent tumor was evaluated twice if follow-up imaging before surgery was available. Local recurrent tumor initially exhibiting distinct signal characteristics compared to the primary tumor demonstrated subsequent changes in signal intensity or contrast-enhancement during follow-up, progressively converging with the features of the primary tumor. Especially the criterion contrast-enhancement increased in more than one-third between time-point 1 and 2. However, eight patients received chemotherapy or radiotherapy in the meantime. Therefore, an influence on contrast-enhancement cannot be disregarded. In only 10 cases, true progression was detected at time-point 1, and 14 patients received no therapy or surgery between time-point 1 and time-point 2. Both the small size of the recurrent tumor and the different signal characteristics compared to the primary tumor may be responsible for this.

It should be noted that our evaluation of the primary tumors and the recurrences is based on qualitative analysis and does not include quantitative measurements, such as radiometric analyses of signal intensity. This limitation is due to the multicentric nature of our cohort, where MRI examinations are conducted in various clinics with different protocols and inhomogeneous sequence techniques, most of which involve sequences of the last two decades, and the availability of 3D sequences was not standard. Future research should aim to incorporate these advanced quantitative methods to validate and extend our findings, aligning with the growing trend towards AI-driven evaluations.

Local recurrent tumors and metastases can differ in signal intensity and contrast-enhancement as already described in a small medulloblastoma cohort of *n* = 17 [30]. Therefore, also valid for ependymal neoplasms, any new lesion at the primary tumor site, regardless of its signal intensity or contrast-enhancement, is suspect for local recurrent tumor.

A study on unenhanced MRI in the follow-up of EPN patients showed suboptimal diagnostic sensitivity [31]. Even if contrast-enhancement may not be substantial for the assessment of a new lesion at the primary tumor site, it is of enormous importance for the assessment of leptomeningeal dissemination. We found M2 mainly contrast-enhancing (78%), therefore, the use of gadolinium on follow-up is inevitable. While there is limited literature on diffusion-weighted imaging in ependymoma metastases, a study by Morana et al. demonstrated that diffusion-weighted MRI is more sensitive than conventional MRI (77% vs. 96%) in detecting metastases in CNS embryonal tumors [32]. M2b showed no contrast-enhancement but restricted diffusion in a small number of three patients in our cohort. This supports the utility of DWI for follow-up in EPN. Interestingly, the configuration of intracranial leptomeningeal dissemination was almost entirely nodular. Only one patient showed laminar leptomeningeal dissemination in addition to M2b. An explanation for this is pending, but a nodular appearance enables much better detectability. Furthermore, M2b appeared similar to the primary tumor in more than one-third of cases. As reported in a recently published cohort [13], most PF-EPN cases exhibited spinal dissemination more frequently (nearly 50%), whereas this was rare in ST-EPN (less than 10%), which predominantly presented with intracranial dissemination. The likelihood of metastasis primarily occurring within the same compartment as the primary tumor seems high. In the case of PF-EPN, the frequent occurrence of M3 disease may also be explained by their characteristic deep localization within the fourth ventricle.

During follow-up, two patients developed diffuse intrinsic pontine glioma after primary PF-EPN. The intervals between the primary and secondary tumors were 23 and 45 months, which is relatively short. Unlike secondary malignancies, post-radiogenic imaging changes in EPN manifest earlier after first-line treatment and decrease after a few months [33]. Thus, time distance from therapy may serve as a criterion to distinguish transient imaging changes from true progressions. It is suggested that proton-beam radiotherapy causes imaging changes more often than photon-beam radiotherapy in EPN patients [33]. Post-radiogenic changes mimicking recurrence were rare and occurred equally after proton and photon radiotherapy. However, we must consider the possibility of selection bias, as our recruitment was primarily from a cohort of confirmed relapses. Another large study on imaging changes after proton beam therapy within the German Brain Tumor Working Group (HIT) is currently ongoing and results are awaited.

We found extraneural metastases only on cranial MRI. Two of the three metastases were near the surgical access. As is known from other tumors, such as craniopharyngioma, the localization fits implantation metastases but histopathological work-up was not performed. Literature lacks a systematic analysis of tumor cell seeding along a surgical tract in EPN patients. As only selected data as cranial and spinal MRIs have been transmitted to the Neuroradiological Reference Center we can only report metastases within the field of these regions. Other reports have found extraneural metastases in the lung, pleura, and lymph nodes due to possible iatrogenic intravascular seeding [34–37]. In a cohort of 81 intracranial and spinal EPN (pediatrics and adults), Newton found two intracranial EPN with extraneural metastases [38]. Even if extraneural metastases are rare, it is important to carefully evaluate the whole MRI, including the localizer.

In the long-term results of the E-HIT-REZ study, Adolph et al. found that the extent of resections was the most important predictor of survival [5]. Massimino et al. and Klawinski et al. described worse survival in patients with symptomatic relapses compared to non-symptomatic EPN patients, where relapse was detected by MRI [17, 39]. These clinical aspects clarify that neuroradiological assessment of initial, postoperative and follow-up MRI is of utmost importance. In addition, in 2022, the RAPNO (response assessment in pediatric neuro-oncology) working-group published a guideline for uniform disease assessment postoperatively and on follow-up, which will be useful for clinical studies, and general clinical practice [40]. With our work, we hope to simplify the categorization and detection of recurrences, especially of local recurrent tumors at small stages, to enable appropriate early therapies and a better re-resectability.

## Conclusion

Recurrences can occur multiple times in EPN patients, and the recurrence patterns differ between ST-EPN and PF-EPN. While PF-EPN predominantly recur locally, ST-EPN are more frequently associated with intracranial dissemination at first relapse. Local recurrent tumors may show differences in signal intensity and less contrast enhancement compared to the primary tumor, without indicating a distinct entity. Leptomeningeal dissemination is rare at diagnosis but becomes more frequent in relapse. Spinal dissemination is common in PF-EPN but not in ST-EPN; the underlying cause for this remains unclear and warrants further investigation. New intraparenchymal lesions after therapy are rare in EPN and were in part transient imaging changes caused by radiation therapy, although we also observed relatively early secondary tumors. The observation that extraneural metastases were predominantly located near surgical accesses raises the possibility of implantation metastases.
